# Material Analysis of Early Medieval Woven Bands from Gródek upon the Bug River and Pień, Poland

**DOI:** 10.3390/ma18235279

**Published:** 2025-11-22

**Authors:** Maria Cybulska, Katarzyna Lech, Irka Hajdas, Jan Sielski, Marcin Wołoszyn, Dariusz Poliński

**Affiliations:** 1 Institute of Architecture of Textiles, Lodz University of Technology, 116 Zeromskiego St., 90-543 Lodz, Poland; 2 Faculty of Chemistry, Warsaw University of Technology, Noakowskiego 3, 00-664 Warsaw, Poland; katarzyna.lech@pw.edu.pl; 3 Laboratory of Ion Beam Physics, ETH Zurich, HPK H25, Otto-Stern-Weg 5, CH-8093 Zürich, Switzerland; 4 Faculty of Process and Environmental Engineering, Lodz University of Technology, Stefanowskiego 2, 90-537 Łódź, Poland; 5 Institute of Archaeology, Department of Medieval and Modern History, University of Rzeszów, Moniuszki 10, 35-015 Rzeszów, Poland; marcinwoloszyn@gmail.com; 6 Leibniz Institute for the History and Culture of Eastern Europe (GWZO), Reichsstraße 4-6, 04109 Leipzig, Germany; 7 Institute of Archaeology, Nicolaus Copernicus University in Toruń, Szosa Bydgoska 44/48, 87-100 Toruń, Poland; dariusz.polinski@umk.pl

**Keywords:** archaeological textiles, metal threads, SEM EDS, HPLC, tandem mass spectrometry, natural dyes, radiocarbon dating, deterioration of textiles, corrosion of silver, Middle Ages

## Abstract

This article presents the results of a comprehensive material analysis of medieval decorative bands from two different excavations in present-day Poland, specifically from early medieval cemeteries in Gródek upon the Bug River and Pień. The bands are complex materials composed of various fibres and precious metals, dyed with natural dyes using recipes that are often unknown today. They represent rare archaeological finds, challenging to analyse not only due to the complexity of their structure and materials but also because of significant deterioration caused by exposure to environmental conditions and harmful substances present in the burial soil. Optical microscopy and scanning electron microscopy (SEM) facilitated the identification of raw materials, manufacturing techniques, and ornamentation. SEM coupled with energy-dispersive X-ray spectroscopy (EDS) was employed to analyse the metal threads, determine their elemental composition, and assess their preservation state. Natural dye identification was performed on selected objects using high-performance liquid chromatography combined with spectrophotometric detectors and tandem mass spectrometry with electrospray ionization (HPLC-UV-Vis-ESI-MS/MS). The analysis of these results enabled drawing conclusions regarding the origin of the bands and their manufacturing methods. The dating of the bands, based on ornamentation and manufacturing techniques, was confirmed by radiocarbon dating, indicating they date back to the 10th–12th centuries. They were produced using two weaving techniques, a narrow haberdashery loom and a tablet loom, primarily from silk and metal threads—silver and silver-gilt. Some materials consisted of red-dyed silk (using kermes or madder), including a metal thread core. The analysis also provided valuable insights into textile degradation, particularly the corrosion mechanisms affecting the metal threads.

## 1. Introduction

Decorative bands woven from silk and metal threads constituted one of the principal techniques, alongside embroidery, for the ornamentation of both secular and liturgical garments during the Early Middle Ages and in subsequent centuries. Archaeological evidence and medieval visual sources indicate that such bands were employed as trimmings along garment openings, hems, and necklines. They also functioned as material for headbands and girdles. Owing to their considerable cost, these decorative elements were accessible exclusively to the upper strata of society. In Eastern, Central, and Northern Europe, they were generally not produced in local workshops but imported, predominantly from Byzantium [1].

Similar plain and patterned bands, dating back to the 11th–13th centuries, can be found in literature and in museum collections. For example, the Victoria and Albert Museum collection contains more than a dozen similar objects, most of them woven on tablets. They can also be found among numerous finds from Russia and Ukraine dating back to the 12th and 13th centuries [1,2,3].

The bands discussed in this publication come from two excavations in present-day Poland: Gródek upon the Bug River and Pień.

The early medieval settlement complex in Gródek upon the Bug River is located at the eastern border of Poland, at the point where the Huczwa River meets the Bug River. Investigations of the cemetery in Gródek were conducted in 1952–1955 as a part of very extensive research on so-called Cherven’ Towns. Many of the finds discovered then, including textiles, were not analysed for various reasons, mainly, due to the enormous amount of found material, a shortage of funds, means and techniques, and the limited availability of comparative material and modern research methods. In 1955 the project was abandoned. Unfortunately, many of the finds were lost. During the dig in 1952–1955, the stronghold’s embankment and the cemetery in the ward were excavated. 466 graves were discovered, with burial goods in some graves (9% of the burials). The grave goods primarily included numerous jewellery, devotional objects, and elements of dress: various types of temple rings, diadems, small bronze buttons, glass bracelets, and finger rings [4]. The textile material includes 23 objects found in a total of 13 graves. Although the area around Gródek is today within the borders of Poland, in the Early Medieval Period (11th–13th centuries) it constituted the western periphery of Kievan Rus’. Poland did not extend its rule over this area until the 14th century. The material culture of this centre also has a decidedly more Ruthenian than Polish character [5,6].

The early medieval cemetery in Pień, dating from the second half of the 10th to the first half of the 11th century, is located in the western part of Chełmno Land. During excavations conducted between 2005 and 2009, 11 chamber graves were discovered, each of them monumental in form and richly furnished with luxurious items, such as an iron axe encrusted with silver, necklace components made of silver and semi-precious stones, including lapis lazuli, a bronze bowl, and textile fragments [7]. The graves had wooden internal structures in the form of a chamber consisting of walls, floor, and ceiling, in which the deceased’s body was placed. In many cases, the deceased’s body was placed in a wooden coffin. Most often, earthen mounds were built over the graves, or wooden structures were erected, forming so-called “houses of the dead”. The individuals buried in these graves belonged to the local elite who lived in a fortified structure with a similar chronology located several hundred meters southwest. Chełmno Land, where Pień is located, occupies a special place in the system of communication, trade, and cultural contacts. It is situated on the border between the lands of the Western Slavs and the Prussians, as well as at the junction of Pomerania, Masovia, and Kuyavia. This region was the intersection of long-distance land routes connecting Ruthenia with Pomerania and water routes along the Vistula River connecting the Baltic Sea with the interior [8,9].

The bands under study exhibit a complex, multi-layered structure and are composed of diverse raw materials and components, including silk, plant fibres, various metals, and dyes, each possessing distinct physicochemical properties and varying resistance to environmental conditions. Some bands have endured nearly 1000 years under the harsh conditions of earthen graves, whereas others have undergone significant deterioration. Consequently, their analysis necessitates the application of multiple analytical techniques to identify the constituent materials and to characterize and better understand the deterioration processes affecting them.

Research on historic textiles employing modern analytical techniques expanded significantly during the 1980s and 1990s [10,11,12,13,14,15]. Initially, these studies focused primarily on textiles from museum collections and were conducted in the context of conservation efforts. Over time, archaeologists increasingly adopted modern material analysis techniques, and with the growing interest in textiles, these methods have become more prevalent in the study of archaeological textile finds [16,17,18,19].

Microscopic and spectroscopic techniques are employed to identify the materials constituting textiles, which is essential not only for selecting appropriate conservation methods but also for understanding the history of material culture. Historical textiles were exclusively manufactured from natural fibres. Protein fibres, such as silk and wool, are readily distinguished using optical microscopy and scanning electron microscopy (SEM) due to their characteristic features: fibre length and thickness, the triangular cross-sectional shape of silk filaments, and the scale patterns on wool fibre surfaces. However, the identification of certain plant fibres requires more advanced techniques, including infrared (IR) spectroscopy, the modified Herzog test, or Raman spectroscopy [17,20,21,22,23,24,25].

In studies of metal threads, elemental composition is most frequently analysed. Various spectroscopic techniques are utilized for this purpose, including energy-dispersive X-ray spectroscopy (EDS), inductively coupled plasma mass spectrometry (ICP-MS), and Raman spectroscopy. These methods also facilitate the examination of metal thread manufacturing techniques by enabling analysis of both surfaces and cross-sections [17,21,26,27,28,29,30].

The modern study of natural dyes in historical textiles commenced with the application of advanced techniques such as high-performance liquid chromatography (HPLC) coupled with UV-Vis detection for the identification of organic colourants [10,12,13]. Subsequent technological advancements, notably the development of atmospheric pressure ionization methods including electrospray ionization (ESI), which can be integrated with mass spectrometry (MS) and tandem mass spectrometry (MS/MS), have substantially enhanced analytical capabilities. These methodologies, readily combined with HPLC, have transformed dye analysis by enabling comprehensive characterization of complex mixtures of colour compounds within textiles, including archaeological specimens [29,31,32,33,34,35,36,37,38,39,40,41,42,43,44,45]. Collectively, these innovations have augmented our capacity to analyse artefacts, reveal intricate dye compositions—many previously unidentified or poorly characterized—and expand understanding of historical dyeing techniques, trade networks, and global economic interactions.

Recent material analysis techniques also encompass strontium isotope analysis, facilitating provenance determination, and micro-computed tomography, permitting non-invasive examination of textiles inaccessible to direct analysis [46,47].

The primary objectives of this research were to analyse the constituent materials, manufacturing, and finishing techniques of the textiles. Additionally, the study aimed to investigate alterations occurring within the archaeological environment, where natural aging and degradation processes transpire. Particular attention was devoted to assessing the influence of material composition, textile manufacturing methods, and environmental factors on degradation mechanisms. Given the substantial deterioration observed in the metal components of the threads, an investigation into their corrosion mechanisms was also undertaken. Consequently, this analysis holds significance not only for historians of material culture but also for contemporary materials science.

## 2. Materials and Methods

**Figure 1:**
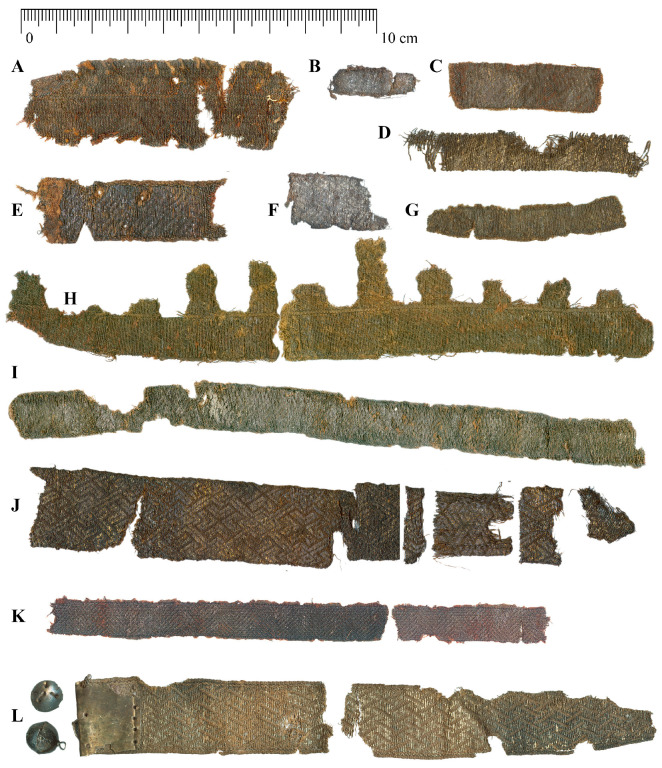
Woven bands form Gródek upon the Bug and Pień (see Table 1).

The textiles from Gródek constitute the largest collection of archaeological silk artefacts from Poland dating to the early Middle Ages. The assemblage comprises 23 silk fabrics, including 11 decorative bands woven from silk and metal threads [48]. Of these, three were recovered from female graves, five from male graves, and the remaining three from graves of indeterminate gender (see Table 1). The bands exhibit significant mineralization, and the fibres are notably brittle. The metal threads have suffered extensive degradation. The sole band in the collection from Pień (Figure 1L) remains in considerably better condition, with metal threads preserved across nearly its entire surface. This preservation is likely attributable to the grave’s construction and distinct soil conditions. The band was discovered in the grave of a small child, positioned near the hips. Presently, the fabrics appear brown, while the preserved metal threads retain a golden hue. Both the fabrics and their components—metal threads and dyes—were subjected to analysis.

All woven bands form Gródek upon the Bug and Pień are illustrated in Figure 1 and characterized in Table 1.

All textiles were scanned at high resolution (1200 dpi) using the HP Scanjet 3970 digital Flatbed Scanner. Based on the scans, a weave analysis was performed and the basic structural parameters were determined: thread count, thickness, and twist. The weave and ornamentation of the fabrics were also analysed [48].

A scanning electron microscope SM-6610LV (JEOL) equipped with an X-Max 80 energy-dispersive X-ray spectroscopy (EDS) analyser (Oxford Instruments, UK) was used for secondary electron image (SEI) observations. SEM examinations were performed at selected points in high vacuum using electron beam acceleration voltages ranging from 10 to 20 kV. SEM analysis allowed for the determination of fibre parameters and identification of the raw material based on fibre morphology. Analysis of metal threads was conducted using Scanning Electron Microscopy (SEM) coupled with Energy Dispersive X-ray Spectroscopy (EDX). It allows for the examination of the surface morphology and elemental composition of the metal components of threads and the identification of corrosion products present on their surface [17,18,28,29,49].

The separation of the colourants was performed using an Agilent 1100 Series High-Performance Liquid Chromatography (HPLC) system. The system was equipped with a Zorbax SB-Phenyl column (4.6 × 150 mm, 3.5 μm, 80 Å) and a Zorbax SB-Phenyl precolumn (4.6 × 12.5 mm, 5.0 μm). The mobile phase consisted of solvent A, 0.15% formic acid in water, and solvent B, methanol. The flow rate was maintained at 0.5 mL·min^−1^, utilizing a gradient elution mode (0 min, 40% solvent B; 15 min, 60% solvent B; 20 min, 70% solvent B; 27 min, 100% solvent B; 35 min, 100% solvent B). Detection was carried out using a combination of spectrophotometric detectors in series—a 1100 Series Variable Wavelength Detector (VWD) set at 280, 330, 550, and/or 600 nm, as well as a 1100 Series Diode Array Detector (DAD) operating across multiple wavelengths (280, 330, 400, 450, 500, 520, and/or 600 nm), capturing spectra from 240 to 650 nm with 2 nm resolution. Additionally, a 6460 Triple Quadrupole Mass Spectrometer with *JetStream* Technology was employed in both negative and positive ionization modes, acquiring data through dynamic multiple reaction monitoring (dMRM) mode [38,40,50]. Additional product ion spectra (MS/MS) were acquired within a mass range from *m*/*z* 50 to *m*/*z*-value of the parent ion + 20 to achieve an upper limit around 20 *m*/*z* above the *m*/*z* of each fragmented ion. The mass spectrometric analyses were managed using MassHunter Workstation software version 7, enabling identification of the colourants in the samples.

The extraction of the colourants from the textile fibres was performed using ultrasonic-assisted extraction followed by centrifugation, according to the procedure proposed by Lech [50]. Specifically, fibres were immersed in 50 μL of a water-methanol-formic acid mixture (9:8:3, *v*/*v*/*v*) and subjected to ultrasonication at room temperature for 30 min, followed by incubation at 60 °C for an additional 30 min. The extracts were diluted with 80 μL of the methanol-water mixture (9:8, *v*/*v*). Additionally, DMSO extracts were prepared similarly, but in this case, the extracts were diluted with 80 μL of methanol.

The chemicals used in the extraction process included LC/MS-grade methanol (from POCH, Gliwice, Poland), LC/MS-grade dimethyl sulfoxide (DMSO; from Fisher Scientific, Branchburg, NJ, USA), and formic acid (from Fisher Scientific, Branchburg, NJ, USA). These solvents and reagents ensured high purity and compatibility with subsequent chromatographic and mass spectrometric analyses.

In order to date the examined collection, radiocarbon dating was performed for selected bands. Samples of the bands were examined under a binocular and transferred to glass vials. The fragile silk fragments were treated with modified acid–base-acid washes [51,52]. The clean silk (mass of ca. 3 mg) was then weighed into Al-cups for combustion in an Elemental Analyser and a subsequent graphitization [53]. Resulting graphite powder was pressed into the Al cathodes for the AMS analysis at MICADAS [54]. Calibration has been performed using OxCal v.4.4.4 software [55] and the INTCAL20 calibration curve [56].

## 3. Results and Discussion

### 3.1. Raw Material and Structure

**Figure 2:**
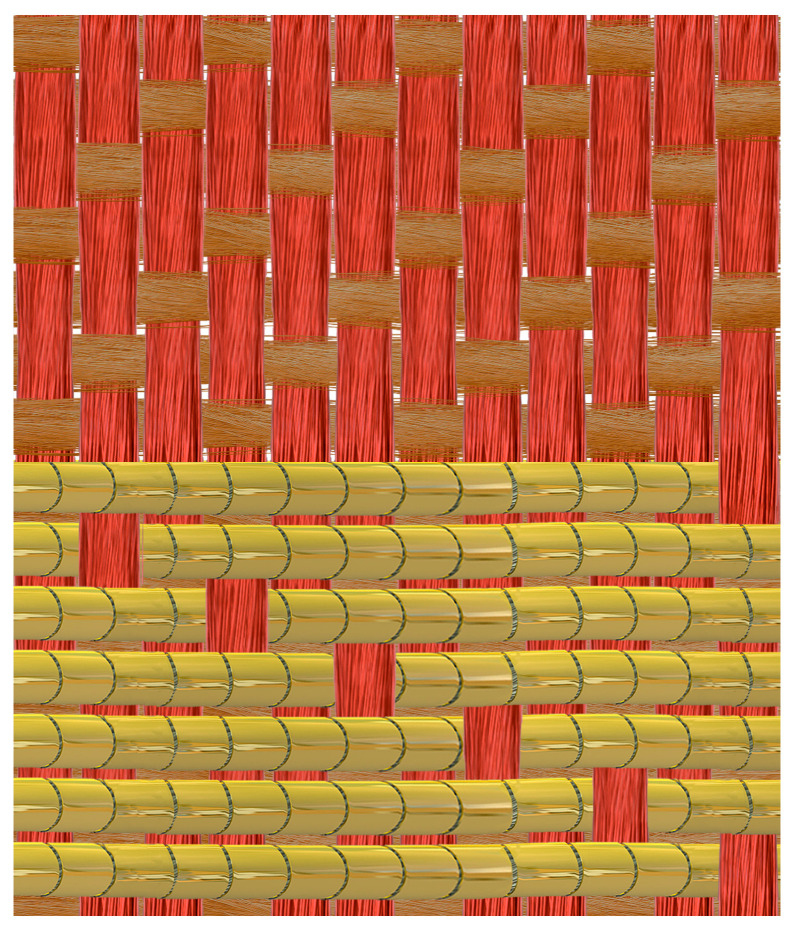
Weft-faced compound twill: red—warp, brown—main weft, gold—supplementary patterning weft.

The analysed bands have a complex, multi-layered structure resulting from the use of weft-faced compound weave, with one warp, main weft and supplementary patterning weft of metal wrapped thread. The first layer consists of a fibrous warp and the main weft in a twill weave. The second layer consists of metal threads bound with warp in a regular twill weave or in various variants of a broken twill weave, creating geometric patterns in the form of interwoven ribbons, rhombuses, and other motifs (Figure 2) [48,57].

Among the examined objects, ten (A–J) were woven on a narrow haberdashery loom, while two bands were woven on a tablets (K and L). The basic structural parameters of the bands, together with descriptions of the weaves and ornamentation, are published in the catalogue of silks from Gródek upon the Bug River [48].

Warp and main weft in all bands are made of silk, what was confirmed by SEM analysis. Natural fibres have a significantly different structure, cross-section and surface characteristics. In the SEM images one can see long smooth, glossy filaments with a diameter of up to 10 µm. Additionally, in a SEM image one can see a cross-section of a fibre with a characteristic triangular shape. This allows for the unambiguous identification of the fibres as silk [17,18].

### 3.2. Metal Threads

Metal threads have been used in textiles since ancient times. They were intended for clothing of the highest social classes: rulers, aristocrats, and high-class liturgical vestments. Initially, they consisted of strips of metal cut from beaten metal sheets. Such flat metal threads are referred to as “beaten and cut” [18,58,59]. Flat metal threads were either applied directly or used to create composite threads by wrapping the strip around a fibrous core, thereby creating a wrapped metal thread. With the improvement of wire production in the Middle Ages, wire began to be used to decorate silk fabrics and haberdashery. A method for producing flat metal thread by flattening a metal filament was also developed, and this type of thread is referred to as “drawn and rolled.” This method was likely not yet in use in the early Middle Ages [58,59].

The distinction between these two types of flat metal threads, in the case of pure gold or pure silver, is determined by examining the strip’s surface and edges. If the edges are slightly rounded and grooves parallel to the thread’s edge, left by the drawing and flattening rollers, are visible, the thread was produced using the “drawn and rolled” method. This difference is even more evident in the case of gilded threads. The gilding process took place before cutting the metal sheet or flattening the wire. When the strip was made from gilded wire, the gold layer is present on both sides. By contrast, a “beaten and cut” strip carries the gold layer only on one side [17,60].

**Figure 3:**
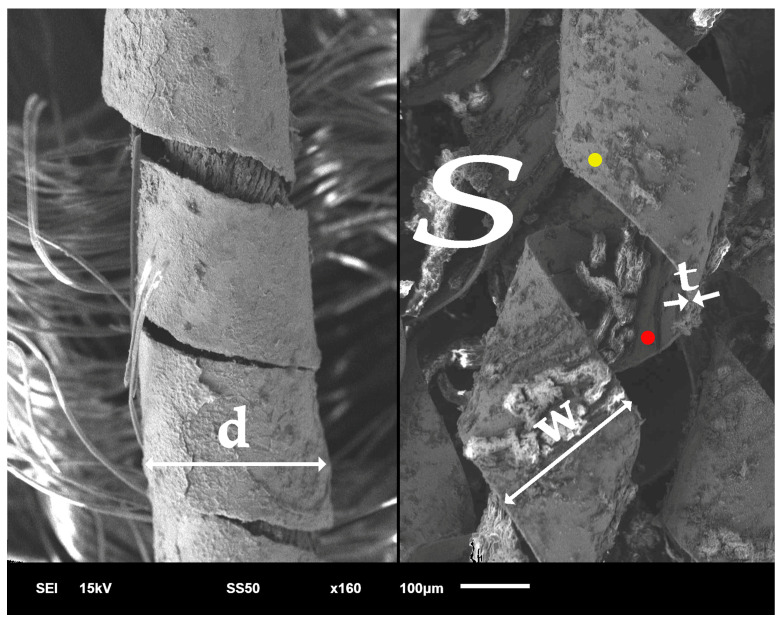
Exemplary metal-wrapped thread and parameters of the thread structure: d—thread diameter, w—width of the metal strip, t—thickness of the metal strip, S/Z—wrapping direction, ●/●—obverse and reverse of the strip.

Metal threads were made from various metals and their alloys, primarily gold, silver, and copper, whose composition could alter the shade of the strip. In the early Middle Ages, however, metal threads were not produced from alloys but from pure gold and silver or gilded silver, although examples of gilded copper threads have also been found [61]. All the threads used in the bands are metal-wrapped threads (Figure 3). They were produced by wrapping a fibre core with a thin strip of metal, with widths (w) ranging from several to several dozen microns and thicknesses (t) from a few to about a dozen microns.

**Figure 4:**
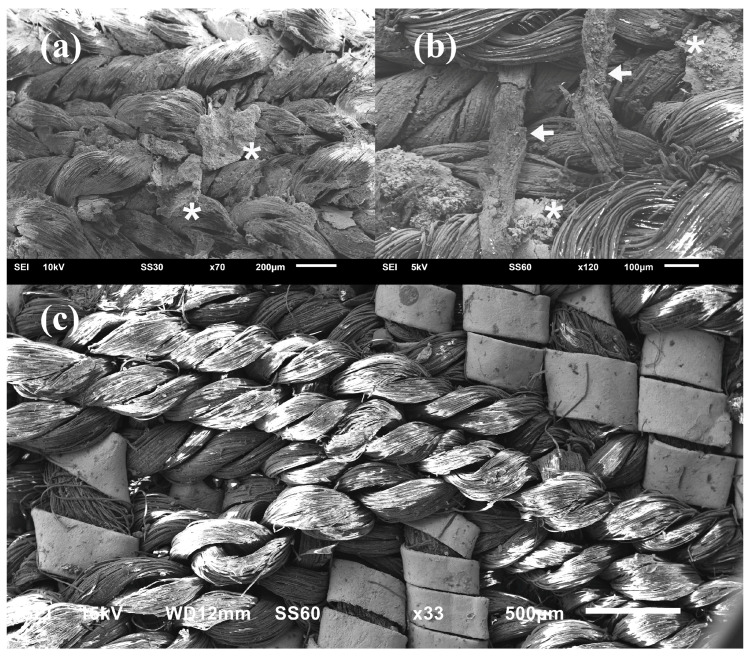
SEM images of bands K (**a**,**b**) and L (**c**). The remnants of the metal strip are marked with asterisks, the remnants of the fibrous core are marked with arrows.

The fibrous cores were spun from various raw materials. Because of the high cost of metal threads, silk cores are most often found in luxury gold and silver threads. However, flax, wool, and even horsehair cores were also used [62]. SEM image analysis revealed that the cores of most metal threads from the examined bands were made of silk. There is one exception: the fibrous core of the thread from band K, which was almost completely decayed, with only small remnants visible in Figure 4a,b. Protein fibres such as silk are known to survive better in acidic environments, while plant fibres are more resistant in alkaline conditions. In most earthen graves and even crypts, clothing made of plant fibres has completely decomposed. The good state of preservation of the warp and weft in the same band suggests that the core is not a protein fibre but a plant fibre [63].

All metal threads were analysed using SEM-EDS. They were observed under SEM and then specific measurement points were selected for further quantitative analysis (Figure S1). Elemental composition measurements were conducted for each thread, determining average percentage weights of two metals—gold and silver—as well as elements potentially related to colouring processes—aluminium and iron—and elements associated with corrosion of metal threads—chlorine and sulphur. The test results are presented in Table 2 which contains mean values of all measurements (total) and mean values of measurements carried out on the reverse side of the metal strip (reverse). Copper, not shown in Table 2, was detected in only six samples, with its content in none exceeding 1% by weight. Full SEM-EDS analysis data are provided in Table S1.

Table 2 shows the mean values of the structural parameters of the metal threads: the diameter of the thread ‘d’, the metal strip width ‘w’ and thickness ‘t’ (Figure 3). All threads were wrapped with a metal strip in the S direction. The diameter of the metal threads varies widely, from 0.21 mm for the strip from band E to 0.44 mm for the band L. The width of the metal strip is more uniform and does not depend on the final thickness of the thread. The thickness of the strip ‘t’ was difficult to measure precisely due to the layer of corrosion products on its reverse, and the values given in Table 2 are only rough estimates. Structural parameters were not determined for the thread from band K due to the almost complete degradation of the metal thread. The thickness of the gold layer of the silver gilt threads was less than 1 µm.

All bands from Gródek (A–K) are made of gilded silver thread with strips cut from gilded sheet. The band L from Pień is made of pure silver.

### 3.3. Deterioration of the Bands

Textiles rarely survive in difficult archaeological environments. Natural fibres, such as silk, are especially susceptible to degradation due to their chemical content, which is a nutrient for microorganisms, such as fungi and bacteria. However, well-preserved objects can often be found. One of the most important factors influencing textile survival is con-tact with metal objects. In the cemetery in Gródek, only textiles made with metal threads survived- small fragments of silk embroidered with metal threads and the bands presented in the article survived from the clothing of the deceased buried there [48,63,64,65,66].

The degree of deterioration of the bands varies greatly. Visually, the band L from Pień and band J from Gródek are the best preserved. Photographs show a pattern created by metal threads, preserved over almost the entire surface of the bands (Figure 1 and Figure 4c). However, in most bands, the metal threads have deteriorated, ranging from partial destruction of the metal strip while maintaining the silk core of the thread to complete degradation of the core and the metal strip, as in band K (Figure 4a,b).

**Figure 5:**
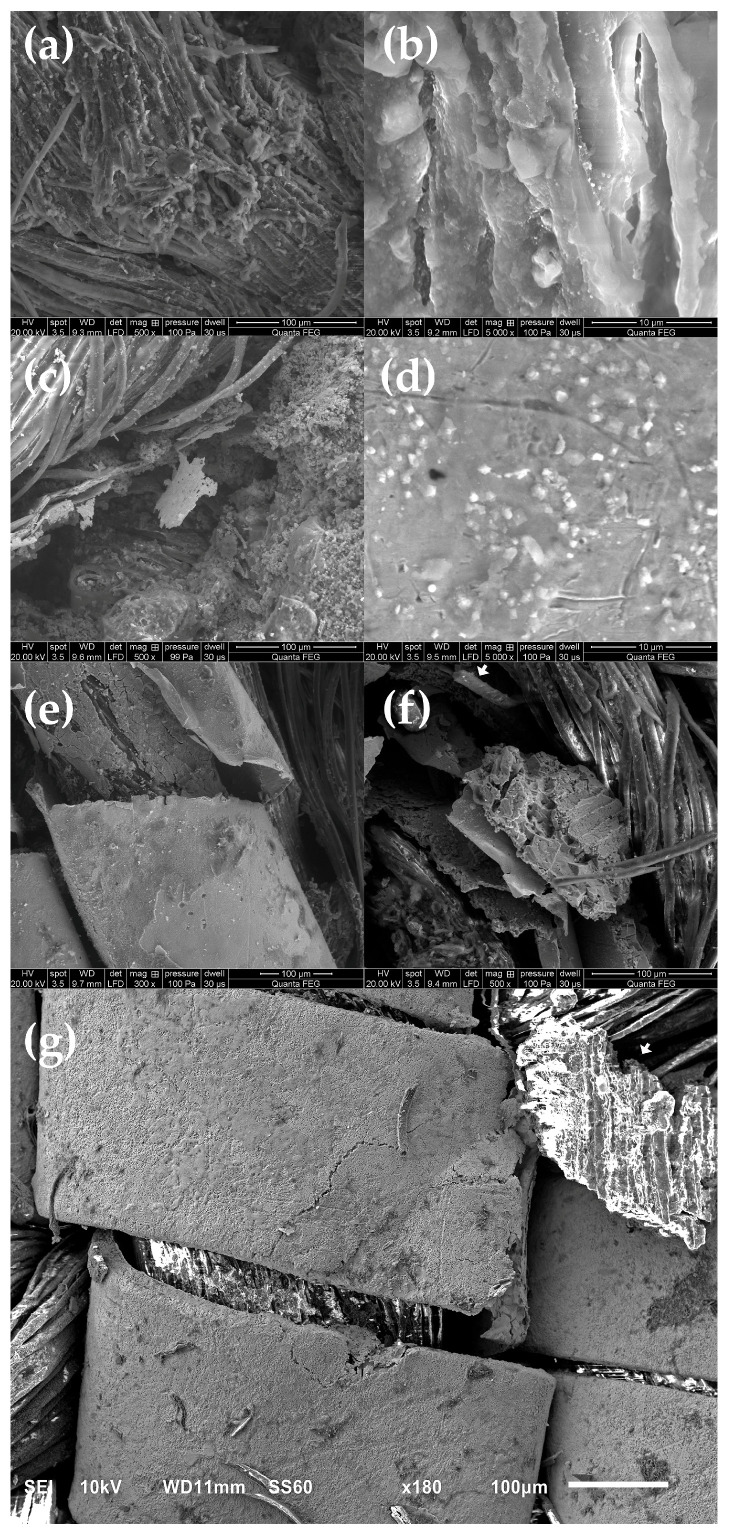
Deterioration of the metal threads: (**a**,**b**) mineralization of fibres—positive pseudomorph; (**c**,**d**) crystalline structures; (**e**) corrosion of metal strip and delamination of the gold leaf; (**f**) corrosion of metal thread: negative pseudomorph on the reverse of the strip and delaminated gold leaf, silk fibre with visible triangular cross-section marked with an arrow; (**g**) well-preserved surface of the silver thread and a fragment of a negative pseudomorph on the reverse of the strip marked with an arrow.

The fibres are weak and brittle and crumble into dust upon manipulation. This fragility results from the natural aging of fibres under archaeological conditions. Silk threads are partially preserved due to mineralization caused by the diffusion of metallic cations from the environment and the metal components of the threads (Figure 5a,b). Textile mineralization is typically associated with the presence of iron and copper; however, in this case, it is also linked to compounds formed as a consequence of silver corrosion. Mineralization leads to the formation of pseudomorphs: negative pseudomorphs occur when the fibres degrade, leaving a mold-like mineral cast of the fibres, whereas positive pseudomorphs arise when the original fibres are replaced by mineral matter that retains the fibres’ shape, often preserving some original organic material [29,63,67,68,69].

The primary cause of deterioration of metal threads is the presence of sulphur and chlorine, which react to form silver chloride (AgCl) and silver sulphide (Ag_2_S) in archaeological environments. Silver generally resists corrosion due to a protective silver oxide patina; however, in chloride-rich environments, silver chloride forms a brittle, finely granular layer on the silver surface. Under corrosive conditions, silver objects may be entirely transformed into AgCl. The elevated chlorine content observed in all tested samples appears to be the principal factor driving corrosion of metal threads in the examined bands [49,70,71]. Silver sulphide forms when silver interacts with hydrogen sulphide, a product of anaerobic bacterial decomposition of organic matter. In burial environments, this compound primarily originates from the decomposition of the interred body. Sulphur was detected in nine of the tested threads. Analysis of the data presented in Table 2 reveals that in six instances, sulphur content is higher on the reverse side of the metal strips. Additionally, sulphur may be released due to the degradation of the silk protein structure, which contains, albeit in minor quantities, sulphur-containing amino acids [72,73].

Figure 5 illustrates various manifestations of metal thread deterioration, including mineralization and positive pseudomorphs retaining the shape of fibres (Figure 5a,b), the development of granular and crystalline structures within the metal thread and on the surface of the metal strip (Figure 5c,d), silver corrosion accompanied by negative pseudomorphs on the reverse side of the strip (Figure 5e–g), delamination of the gold layer (Figure 5e,f), as well as fragmentation of both the metal strip and silk fibres. It is evident that corrosion initiates internally within the threads. For threads featuring a gold layer on the surface, analyses reveal that in the majority of cases, nearly all silver has undergone corrosion, resulting in brittleness and porosity, while the gold leaf’s integrity is preserved to varying degrees. The brittleness of silver chloride contributes to the overall fragility of the thread, causing cracking and fragmentation of the silver strip itself and, consequently, the gold layer. A comparable degradation mechanism is observed in the relatively well-preserved silver thread from band L. The fragment of the deteriorated silver strip depicted in Figure 5g exhibits corrosion on the reverse side and an AgCl negative pseudomorph that mirrors the silk fibres of the core. These observations underscore the necessity for comprehensive examination prior to initiating textile conservation procedures to prevent damage to objects that may appear visually intact.

The corrosion mechanism is influenced by multiple factors. Initially, a patina develops on the more exposed outer surface of the metal strip, serving as a protective barrier against corrosion. This phenomenon predominantly affects silver threads, which exhibit higher corrosion resistance compared to gilded threads [49,71].

Conversely, the reverse side of the metal strip (silver) adheres to the fibre core, which possesses a high capacity to absorb water and aqueous solutions of harmful chemical compounds, potentially leading to their increased concentration. It is also noteworthy that the cemetery in Gródek is situated on loess soils, which exhibit significantly higher water sorption compared to the podzolic soils near Pień [4].

### 3.4. Dyes and HPLC-UV-Vis-ESI-MS/MS Analysis

The study of dyes in textiles necessitates meticulous sample selection for analysis. Fabrics are made from several different sets of threads: one or two warps and several wefts, each potentially dyed with different dyes to achieve the desired ornamentation. Therefore, separate samples must be prepared for each group of threads. This process is relatively simple in well-preserved textiles. Archaeological fabrics, however, present many challenges, primarily due to the fragility of the fibres, which makes it difficult to separate the individual components. Regardless of their original colours, the threads now appear in various shades of brown, making it hard to distinguish them. Another significant issue is the advanced deterioration and mineralization, which cause the fibres to become stiff and brittle. In some cases, separating individual threads is impossible without completely destroying the material.

For these reasons, four bands (A, H, I, and K) were selected for dye analysis. This choice was guided by micrograph observations of the objects, where some threads appeared red when unravelled.

To characterize the dyes, a comprehensive analytical approach using HPLC-UV-Vis-ESI-MS/MS was applied to micro-samples (≤0.2 mg) from textile bands, including warp threads, weft threads, and metal thread cores. The extracted colourants were separated and analysed in dynamic MRM mode for both negative and positive ions, across multiple wavelengths. This approach provided profiles of the organic dyes and revealed the technological diversity of the fabric.

**Figure 6:**
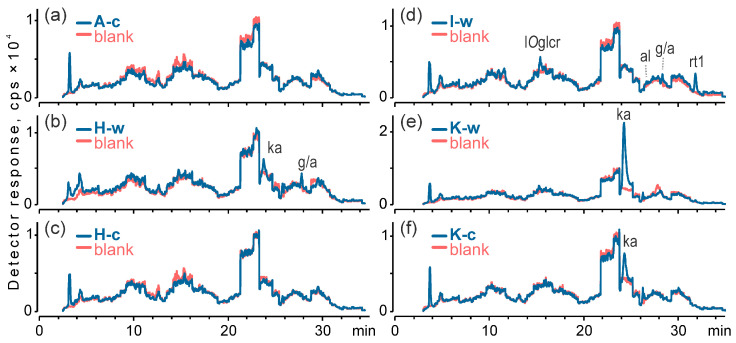
TIC chromatograms acquired by ESI-MS/MS in dMRM mode of negative ions for methanol-water-formic acid blanks and sample extracts from: (**a**) band A (core), (**b**) band H (warp/weft), (**c**) band H (core), (**d**) band I (warp/weft), (**e**) band K (warp/weft), and (**f**) band K (core); l*O*glcr—luteolin *O*-glucuronide, ka—kermesic acid, al—alizarin, g/a—genkwanin/acacetin, rt1—anthraquinone dimer; w—warp and main weft; c—fibrous core of metal thread.

Given that these were micro-samples of delicate, decaying, and severely damaged archaeological threads, all extracts were essentially colourless, and the chromatograms contained very few natural dye markers (Figure 6). Only a handful of small peaks were observed, mostly corresponding to the primary colouring compounds present in the dyes. Such low concentrations of colouring compounds had been previously reported in archaeological textiles recovered from medieval graves [74]. In both cases, identifying organic dyes in the threads was only possible using a mass spectrometer with a targeted detection approach. This method is much more sensitive to most dyes than spectrophotometric detectors. Relying solely on spectrophotometry would have been insufficient for effective dye identification.

Across the chromatograms acquired with the ESI MS/MS detector in negative ion mode, a key peak associated with kermesic acid was found in three samples, H-w, K-w, and K-c. This peak was most intense in K-w and least in H-w. The presence of kermesic acid without detectable carminic acid suggests the use of kermes, a red dye of animal origin derived from the scale insect *Kermes vermilio* Planchon.

Kermes is one of the oldest known dyes, with origins tracing back to ancient times across the Mediterranean, the Near East, and Asia—it was traded along the Silk Road [75,76]. Due to its high cost, kermes was mainly used for dyeing luxury garments. In the Middle Ages, after the knowledge of ancient purple dyeing with murex snails was lost, kermes gained even greater importance. It played a significant role in medieval European trade and became one of the most valuable dyes, used to colour the finest silk and wool textiles [76].

Kermes has been identified in many medieval textiles, including garments of rulers and nobility, Celtic fabrics [76], Danish Viking Age textiles [77], and Hispano-Moorish samite textiles [76]. Its vibrant colour and rarity made it a symbol of wealth and prestige throughout history.

Furthermore, the results revealed that one sample (I–w) contained a distinct peak of an anthraquinone dimer (*m*/*z* 491 [M–H]^−^, rt1), a compound previously noted as a dye component in madder [38] and detected in numerous historical samples [38,43,78]. Although its MS/MS spectra have been published [38], the proposed structures [38,78] have not been verified or unambiguously confirmed. Nonetheless, the presence of this dimer, especially alongside traces of alizarin, indicated that the this thread was dyed with roots of madder species, possibly *Rubia tinctorum* L. Additionally, this sample contained traces of luteolin *O*-glucuronide, which may suggest the use of a yellow flavonoid dye based on luteolin.

Madder has been used since ancient times, serving as one of the earliest and most popular dyes in Egypt, Greece, and Rome [75,76]. Its use persisted beyond the fall of the Roman Empire, continuing to be widely cultivated and utilized throughout Europe during the early Middle Ages [76]. Historically, various species of the *Rubia* genus were collectively referred to as madder, but the primary source of true red was *Rubia tinctorum* L. [75]. It was mainly employed for dyeing wool, with silk being less commonly dyed.

Based on the results of HPLC-UV-Vis-ESI-MS/MS analysis, it is difficult to definitively identify the exact dye source. Nevertheless, the presence of traces of alizarin suggests the possible use of *Rubia tinctorum* L. [75,76].

All results of the dye analysis are summarized in Table 3.

The results indicate that three out of four examined bands were produced using red-dyed silk. The incorporation of dyed threads significantly increased the textile’s value—by 50% to over 100%—compared to undyed silk [79]. The most remarkable discovery, however, was that the core of one metal thread (sample I-c) was also dyed red. This is quite exceptional, as metal thread cores in the medieval period were typically left undyed (in silver threads) or dyed yellow (in gold threads). An exception was made for the so-called Cypriot gold threads, which were made from gilded membranes such as leather, animal gut, or paper. These membranes were cut into strips, wound around a fibrous core, and often dyed red for use in embroidery [59,80,81].

Moreover, in the chromatograms acquired using an ESI MS/MS detector in positive ion mode, most extracts showed only traces of isatin, with no peaks corresponding to indigotin. Therefore, it was concluded that these traces were not directly associated with the use of indigo or woad on the examined fibres.

**Figure 7:**
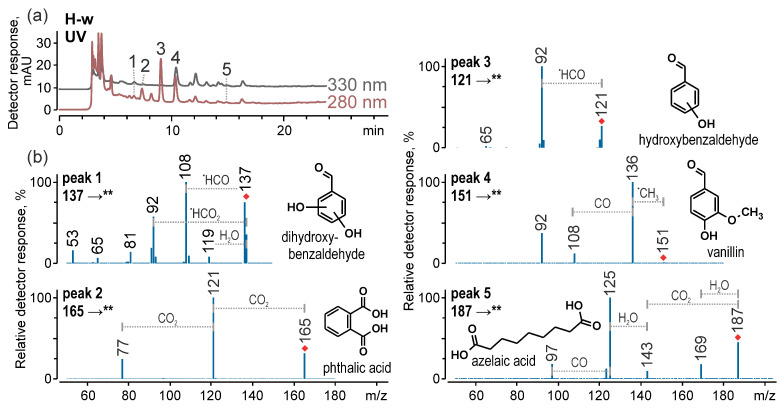
Chromatograms obtained with a spectrophotometer at 280 nm and 300 nm for the methanol-water-formic acid extract of sample H-w (**a**), and product ion spectra (MS/MS) acquired in negative ion mode for compounds eluting as peaks 1–5 (**b**).

Despite the limited number of peaks corresponding to the colourants in the MS/MS chromatograms, several intense signals were detected in chromatograms acquired with a spectrophotometric detector at 280 nm and 330 nm (Figure 7a). The subsequent analysis of their negative ion MS/MS spectra enabled the identification of most of these compounds, including dihydroxybenzaldehyde (*m*/*z* 137 [M–H]^−^, 6.7 min), phthalic acid (*m*/*z* 165 [M–H]^−^, 7.6 min), hydroxybenzaldehyde (*m*/*z* 121 [M–H]^−^, 9.2 min), vanillin (*m*/*z* 151 [M–H]^−^, 10.4 min), and azelaic acid (*m*/*z* 187 [M–H]^−^, 14.8 min). The MS/MS spectra and their interpretations are shown in Figure 7b. According to the authors’ practical experience, these substances are commonly found in historical textiles, especially archaeological samples, likely resulting from the degradation of fibres and dyes influenced by environmental conditions and microbial activity. The identities of phthalic acid and vanillin were later confirmed through comparison with standards, while the identification of azelaic acid was verified by matching its MS/MS spectrum with data available in published literature [82].

### 3.5. Radiocarbon Dating

Two bands, F and I, were submitted for 14C analysis (see Figure 1 and Table 1). The samples were treated with solvents in a Soxhlet apparatus [83].

The standard Acid–Base–Acid (ABA 60) treatment used in the radiocarbon laboratory was modified for fragile silk and was monitored to observe the condition of the samples. Chemical treatment resulted in separation of the metal fragments, which turned out to be gold leaf fragments, as confirmed by SEM EDS analyses (Table 2). The infrared spectra of clean samples confirmed the purity of silk.

**Figure 8:**
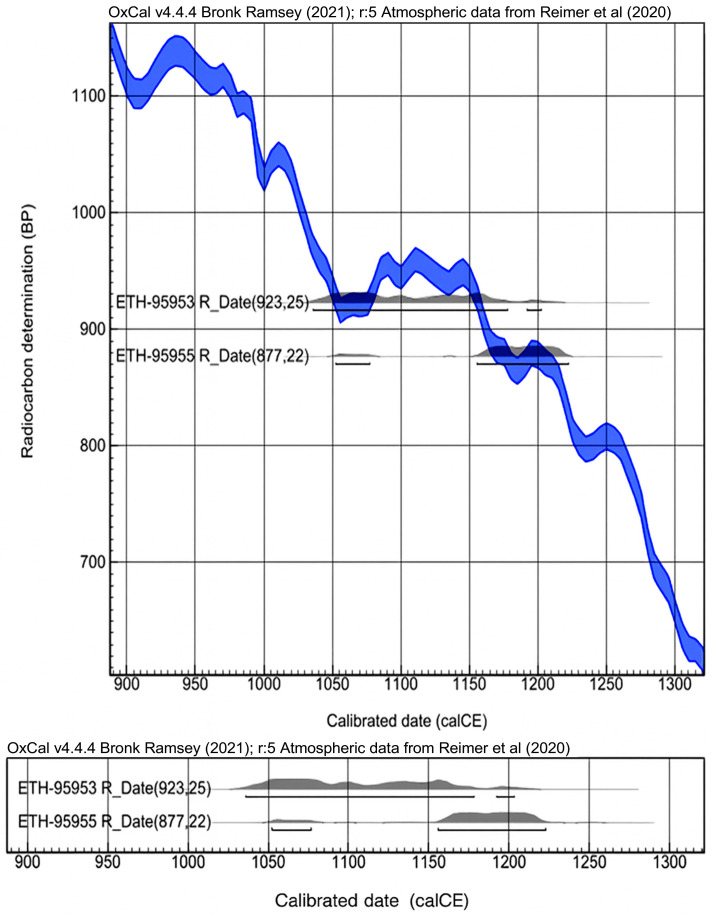
Calibrated radiocarbon ages plotted on the calibration curve: bands I (ETH-95955) and F (ETH-95953).

The first radiocarbon ages obtained on material from the 20th century excavations confirmed that the textiles were produced in 11th–12th centuries CE, as based on the textile analysis (see Table 4) [48]. The radiocarbon dates are plotted on the calibration curve presented in Figure 8.

No ^14^C dating was performed for the L band from Pień. However, radiocarbon dating of the bones from grave 40 in Pień, where the band was found, were as follows: 1085 ± 30 BP [84], and after calibration 892–1021 CE (σ2 range; 95.4%).

Based on radiocarbon dating, it can therefore be concluded that the entire collection of bands examined dates from the 10th–12th centuries.

## 4. Conclusions

The integrated analysis of early medieval silk and metal thread bands from Poland demonstrates the profound potential of material analysis methods and interdisciplinary research in unravelling the complexities of archaeological textile artefacts. The detailed examination of raw materials, manufacturing techniques, and environmental factors has provided valuable insights into the technological sophistication of these luxury items. The identification of silk fibres, silver and gilded silver threads, and valuable dyes underscores the high craftsmanship and access to luxury materials by the elite classes of the period.

Corrosion of metals under archaeological conditions has been extensively studied; however, the corrosion of composite metal threads in archaeological and museum textiles remains poorly understood. On one hand, metal components contribute to the preservation of fibres, but on the other hand, products of fibre degradation may serve as additional sources of corrosion for the metal components. The primary cause of corrosion in metal threads is chlorine, which transforms silver strips into silver chloride. Although silver chloride retains the external form of the thread, it is highly brittle, leading to fragmentation of both the strip and the gold leaf on its surface. Notably, the corrosion mechanism appears to progress from the interior of the thread, likely due to sorption properties of the fibrous core. This phenomenon warrants further investigation on a broader scale, employing complementary analytical methods to enable phase identification of corrosion products and to enhance understanding of the degradation mechanisms affecting metal threads. Such studies are planned by the authors for future research.

Compared to metal components, silk fibres were relatively well preserved, thanks to the presence of silver, which has antifungal and antibacterial properties, and to partial mineralization. However, this resulted in their high brittleness, and in some cases, the object could not be manipulated without risking destruction.

Understanding the complex deterioration mechanisms affecting archaeological textiles, particularly the mineralisation of silk and the corrosion processes of metal threads, is critically important for their conservation and preservation. Notably, even metal threads that appear well preserved can become extremely fragile and susceptible to damage during conservation treatments. This underscores the necessity for meticulous, non-invasive analyses prior to any intervention. Detailed investigations are essential to accurately assess the condition of these objects and to develop tailored conservation strategies that effectively safeguard their structural integrity.

The study of natural dyes used on selected silk threads highlights the challenges of analysing archaeological textiles, primarily due to extensive deterioration and mineralisation of both the fibres and dyes, as evidenced by abundant peaks of degradation products in chromatograms. Despite these obstacles, advanced mass spectrometry techniques have enabled the identification of key natural dyes such as kermes and madder, indicating the use of luxury dyes in medieval bands. Additionally, the discovery of a red-dyed metal thread core underscores the technological complexity and historical significance of these objects. Overall, the findings demonstrate the essential role of sensitive analytical techniques, such as HPLC-UV-Vis-ESI MS/MS, in revealing historic dyeing practices and material culture, even in heavily degraded samples.

Radiocarbon dating of the two examined silk bands suggests that they date from the 10th to the 12th centuries. The results were consistent, although the dates fall within a complex calibration range. This finding aligns with the results of material analyses concerning the weaving technique and the style of fabric ornamentation.

**Figure 9:**
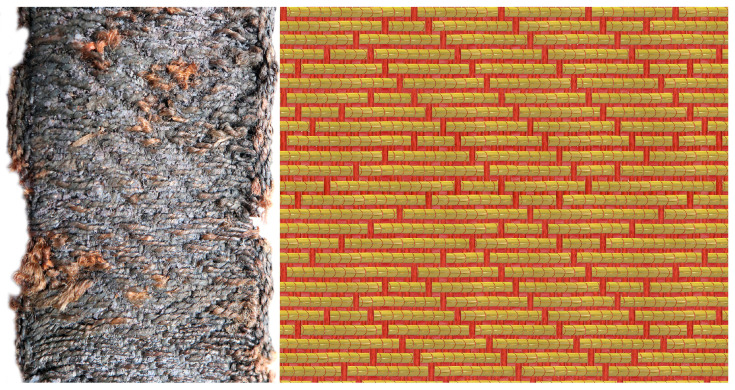
Photograph of a fragment of tape I from Gródek and a visualization of its original appearance based on research results.

Advanced analytical methods such as SEM, EDS, HPLC, and radiocarbon dating have proven indispensable in revealing both the material composition and the visual appearance of these bands, enabling reconstructions of their original appearance and technological features (Figure 9). These techniques not only deepen our understanding of medieval textile technology and dyeing practices but also expand scientific knowledge concerning material aging and corrosion in the archaeological context.

Despite the vast potential of material analysis methods, there are certain limitations and conditions for their application. Archaeological textiles are highly specific, and the research methodology differs fundamentally from that used for contemporary materials. Above all, they are extremely heterogeneous—partly due to their manufacturing techniques, but primarily because of their advanced state of deterioration. For organic dyes, this is reflected in the small amount of colouring compounds that remain in samples; for metal threads- in changes to their morphology and composition. A standard approach can easily overlook areas—sometimes very small—that contain crucial information about the object under examination. Therefore, before and during each study, a thorough inspection of the samples is essential, along with close collaboration between material scientists and historic textile specialists. Given the unique nature of these objects, it is also vital to employ the least destructive sampling and analytical methods available. 

## Figures and Tables

**Table 1:** Woven bands form Gródek upon the Bug and Pień.

Fabric Code	Visual Description of the Fabric	Inv. No	Grave	Gender
A	gold patterned with embroidery	Gródek 153/55	423	female
B	gold unpatterned	Gródek 243a/55	466	male
C	gold patterned	Gródek 130/55	416	female
D	gold unpatterned	Gródek 94/55b	389	male
E	gold patterned	Gródek 152/55c	421	?
F	gold patterned	Gródek 530/54	230	male
G	gold unpatterned	Gródek 493/54	254	?
H	gold patterned	Gródek 195/53	80	?
I	gold patterned	Gródek 96/55a	368	female
J	gold patterned	Gródek 529/54	230	male
K	gold patterned, tablet woven	Gródek 54/55	379	male
L	gold patterned, tablet woven	Pień 35/07 B	40	child—male

**Table 2:** Elemental composition and structural parameters of metal threads from SEM-EDS analysis.

Fabric Code	Measurement Points	Weight %						d mm	w mm	t μm
		Cl	S	Al	Fe	Ag	Au			
A	total	6.10	8.31	10.03	3.51	58.90	13.15	0.22	0.18	---*
	reverse	5.45	13.78	14.44	25.21	41.12	0.00			
B	total	19.92	2.00	1.78	1.45	72.02	2.83	0.25	0.26	---*
	reverse	23.51	1.10	0.80	0.00	74.59	0.00			
C	total	0.76	0.00	1.03	0.71	27.67	69.83	0.33	0.31	11.00
	reverse	6.22	0.00	9.33	8.44	76.00	0.00			
D	total	7.59	0.00	0.73	0.00	23.53	68.15	0.35	0.31	17.00
	reverse	24.44	0.00	0.44	0.00	75.12	0.00			
E	total	15.77	0.43	0.71	0.38	47.80	34.91	0.21	0.24	10.00
	reverse	20.47	0.00	4.72	0.00	74.80	0.00			
F	total	21.96	1.22	0.58	0.00	73.63	2.61	0.26	0.27	---*
	reverse	23.51	1.10	0.80	0.00	74.59	0.00			
G	total	19.50	0.90	0.65	0.21	62.52	16.23	0.31	0.31	12.00
	reverse	15.39	4.94	0.61	0.00	79.05	0.00			
H	total	1.10	0.00	0.72	0.00	29.44	68.74	0.27	0.34	10.00
	reverse	3.36	1.56	0.74	0.00	94.34	0.00			
I	total	6.29	0.12	2.22	0.73	31.07	59.57	0.38	0.30	9.00
	reverse	9.92	5.69	13.77	7.89	62.74	0.00			
J	total	13.12	0.00	0.62	0.00	40.77	45.49	0.27	0.24	11.00
	reverse	25.43	0.00	0.59	0.00	73.98	0.00			
K	total	11.55	5.01	4.31	7.93	61.99	9.21	---*	---*	---*
	reverse	15.04	0.33	1.79	18.20	64.64	0			
L	total	0.10	1.60	0.39	1.00	96.91	0.00	0.44	0.21	15.00
	reverse	1.20	12.20	20.40	18.20	48.00	0.00			

* due to the high degree of destruction, the measurement was not performed.

**Table 3:** Compounds and dyes identified in thread samples from woven bands found at Gródek upon the Bug River.

Fabric Code	Sample	Primary Compounds	Trace Compounds	Probable Dye	Colour
A	c	---	---	N/A	N/A
H	w	---	kermesic acid, genkwanin/acacetin	kermes (*Kermes vermilio* Planchon)	red
H	c	---	---	N/A	N/A
K	w	rt1 (anthraquinone dimer)	alizarin, luteolin *O*-glucuronide, genkwanin/acacetin	madder specie (maybe *Rubia tinctorum* L.) + yellow flavonoid dye	red or orange
I	w	kermesic acid	---	kermes (*Kermes vermilio* Planchon)	red
I	c	kermesic acid	---	kermes (*Kermes vermilio* Planchon)	red

w—warp and main weft; c—fibrous core of metal thread.

**Table 4:** Results of ^14^C dating.

Lab No. ETH-	Fabric Code	Grave	Material	^14^C Age ± 1σ (BP)	Calibrated Age (2σ Range; 95.4%)
95953	F	230	silk and gilded silver	923 ± 25	1036–1203 CE
95955	I	368	silk and gilded silver	877 ± 22	1052–1222 CE

## Data Availability

The original contributions presented in this study are included in the article/Supplementary Material. Further inquiries can be directed to the corresponding author.

## Supplementary Materials

The following supporting information can be downloaded at: https://www.mdpi.com/article/10.3390/ma18235279/s1. Figure S1: SEM images of the band L with the positions of selected EDS measurement points marked for compositional analysis and Table S1: Total elemental composition of metal threads from SEM-EDS Analysis.

## Abbreviations

The following abbreviations are used in this manuscript:

ABA	Acid–Base–Acid treatment
Ag2S	Silver sulphide
AgCl	Silver chloride
BP	Before Present
CE	Common Era
DAD	Diode Array Detector
dMRM	Dynamic Multiple Reaction Monitoring
DMSO	Dimethyl sulfoxide
EDS	Energy-Dispersive X-ray Spectroscopy
HPLC	High-Performance Liquid Chromatography
MS	Mass Spectrometry
SEM	Scanning Electron Microscope
UV	Ultraviolet radiation
Vis	Visible radiation
VWD	Variable Wavelength Detector
