# Supplementary material for: Material Analysis of Early Medieval Woven Bands from Gródek upon the Bug River and Pień, Poland
Source: Materials (Basel). 2025 Nov 22;18(23):5279. doi: 10.3390/ma18235279 (PMC12692763; doi:10.3390/ma18235279)
Supplement: Supplementary file 1 [file materials-18-05279-s001.zip › materials-3985126-supplementary.pdf]

Supplementary Materials

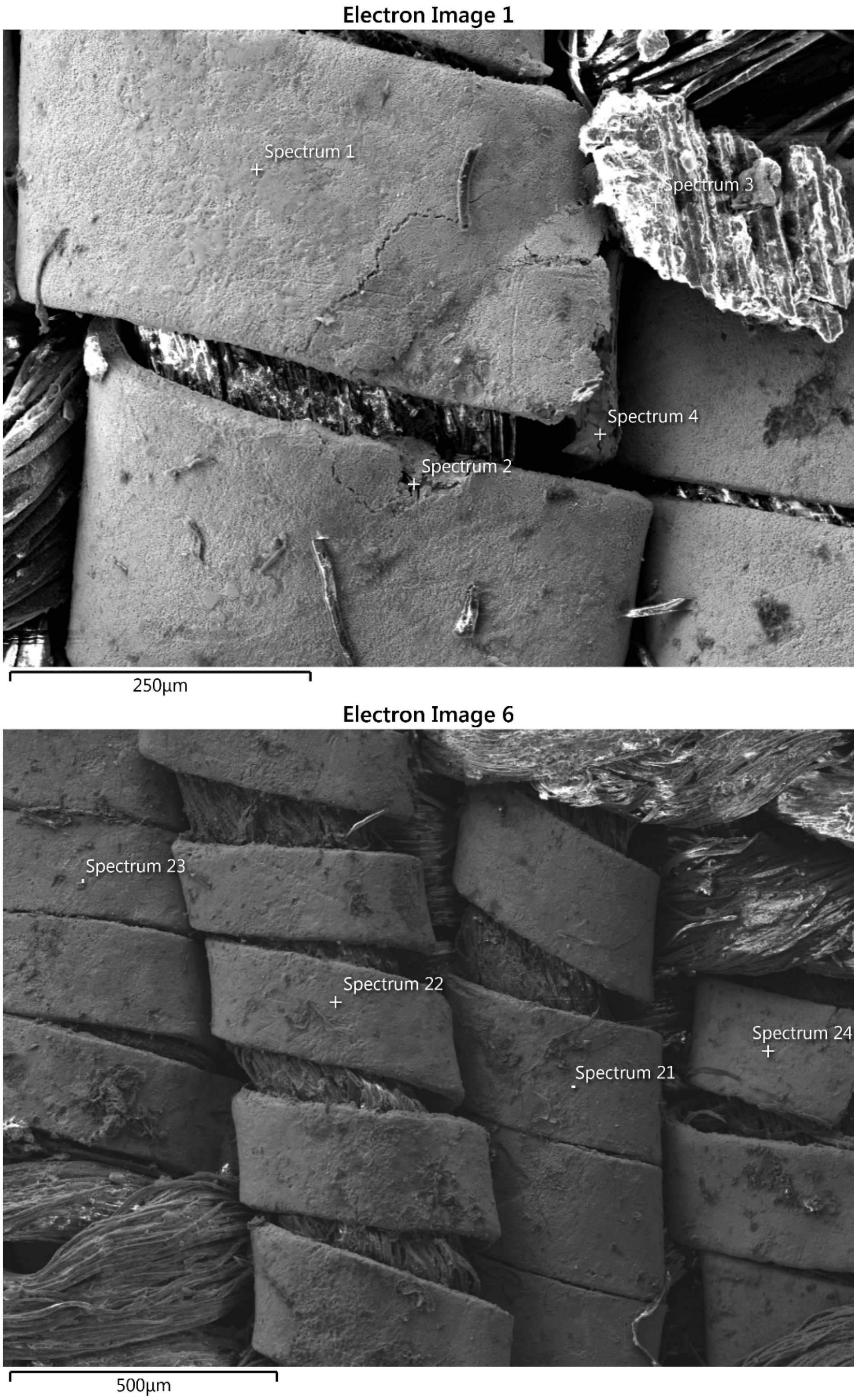

Figure S1. SEM images of the band L with the positions of selected EDS measurement points marked for compositional analysis.

Table S1. Total elemental composition of metal threads from SEM-EDS analysis.

Fabric code	Weight %											
	C	N	O	Ca	Si	Cl	S	Al	Fe	Cu	Ag	Au
A	33,80	8,99	23,77	2,49	6,99	1,44	1,96	2,37	0,83	0,38	13,89	3,10
B	32,92	11,46	17,87	1,09	1,37	7,03	0,71	0,63	0,51	0,00	25,42	1,00
C	17,94	7,46	8,58	0,38	0,00	0,50	0,00	0,67	0,46	0,56	18,01	45,45
D	18,82	7,14	7,33	0,08	0,27	4,97	0,00	0,48	0,00	0,97	15,39	44,56
E	19,67	8,51	6,31	0,18	0,29	10,26	0,28	0,46	0,24	0,00	31,09	22,70
F	19,42	0,00	7,50	0,00	0,00	16,05	0,89	0,42	0,00	0,00	53,81	1,91
G	20,18	4,54	7,15	0,11	0,17	13,23	0,61	0,44	0,14	0,00	42,42	11,01
H	16,17	6,08	9,25	0,94	0,06	0,74	0,00	0,48	0,00	0,86	19,62	45,81
I	18,24	19,58	5,11	7,37	2,50	2,92	0,06	1,03	0,34	0,78	14,42	27,65
J	13,32	5,72	1,66	0,11	0,12	10,37	0,00	0,49	0,00	0,00	32,23	35,97
K	31,64	0,00	17,75	0,75	3,11	5,96	1,55	1,57	3,82	0,00	30,91	2,96
L	12,98	0,16	8,52	0,45	0,62	0,08	1,22	0,30	0,76	0,96	73,97	0,00
